# Chromatin structure and evolution in the human genome

**DOI:** 10.1186/1471-2148-7-72

**Published:** 2007-05-09

**Authors:** James GD Prendergast, Harry Campbell, Nick Gilbert, Malcolm G Dunlop, Wendy A Bickmore, Colin AM Semple

**Affiliations:** 1 Colon Cancer Genetics Group, Division of Oncology, University of Edinburgh, Western General Hospital, Crewe Road, Edinburgh, EH4 2XU, UK; 2 MRC Human Genetics Unit, Western General Hospital, Crewe Road, Edinburgh, EH4 2XU,UK; 3 Public Health Sciences, Department of Community Health Sciences, University of Edinburgh, Edinburgh, UK

## Abstract

**Background:**

Evolutionary rates are not constant across the human genome but genes in close proximity have been shown to experience similar levels of divergence and selection. The higher-order organisation of chromosomes has often been invoked to explain such phenomena but previously there has been insufficient data on chromosome structure to investigate this rigorously. Using the results of a recent genome-wide analysis of open and closed human chromatin structures we have investigated the global association between divergence, selection and chromatin structure for the first time.

**Results:**

In this study we have shown that, paradoxically, synonymous site divergence (dS) at non-CpG sites is highest in regions of open chromatin, primarily as a result of an increased number of transitions, while the rates of other traditional measures of mutation (intergenic, intronic and ancient repeat divergence as well as SNP density) are highest in closed regions of the genome. Analysis of human-chimpanzee divergence across intron-exon boundaries indicates that although genes in relatively open chromatin generally display little selection at their synonymous sites, those in closed regions show markedly lower divergence at their fourfold degenerate sites than in neighbouring introns and intergenic regions. Exclusion of known Exonic Splice Enhancer hexamers has little affect on the divergence observed at fourfold degenerate sites across chromatin categories; however, we show that closed chromatin is enriched with certain classes of ncRNA genes whose RNA secondary structure may be particularly important.

**Conclusion:**

We conclude that, overall, non-CpG mutation rates are lowest in open regions of the genome and that regions of the genome with a closed chromatin structure have the highest background mutation rate. This might reflect lower rates of DNA damage or enhanced DNA repair processes in regions of open chromatin. Our results also indicate that dS is a poor measure of mutation rates, particularly when used in closed regions of the genome, as genes in closed regions generally display relatively strong levels of selection at their synonymous sites.

## Background

Regions of open and closed chromatin structure have recently been defined across the human genome [1]. Gilbert et al showed that regions of open chromatin are often gene dense and appear to correlate well with clusters of broadly expressed genes. They suggested that open chromatin fibre domains provide a chromatin environment more conducive to transcriptional activation. However, many genes are also found in regions of closed chromatin structure. This raised the question as to why would genes be maintained in closed chromatin if this meant they were simply less accessible for transcription. One possibility is that they need to be subject to especially tight transcriptional regulation, and that their aberrant or leaky expression in inappropriate cells cannot be tolerated. However, it has also been proposed that open chromatin structure may make the underlying DNA sequence more susceptible to DNA damage [2].

Although some studies have predicted that rates of mutation are relatively constant across mammalian genomes, analysis of human-mouse alignments has suggested that there may be as much as a 3-fold difference in substitution rates across chromosomes [3], with regions containing genes involved in extracellular communication displaying unusually high levels of synonymous substitutions [4]. Previous studies have also shown that, in mammals, genes within close genomic proximity undergo similar rates of neutral divergence and evolution [4-6]. For example, Williams and Hurst showed that the mean difference between the Ka values (substitution rate at non-synonymous sites) of 176 pairs of linked genes was significantly lower than would be expected by chance [5]. Similar results were also observed with Ks (substitution rate at synonymous sites) and Ka/Ks (often used to infer the mode and strength of selection). Consequently they proposed that the murid genome was split into domains of evolution. The reason for this was unknown, but it is possible that some aspect of chromatin structure over different genomic regions influences the rate of DNA damage or its repair.

The availability of a map of long-range chromatin structure across the human genome [1] allows us to assess this idea and, through the comparison of various measures of neutral variation, we have identified those forms of chromatin structure associated with the highest rates of background mutation.

## Results and discussion

### Non-dS measures of mutation are highest in closed chromatin

**Figure 1:**
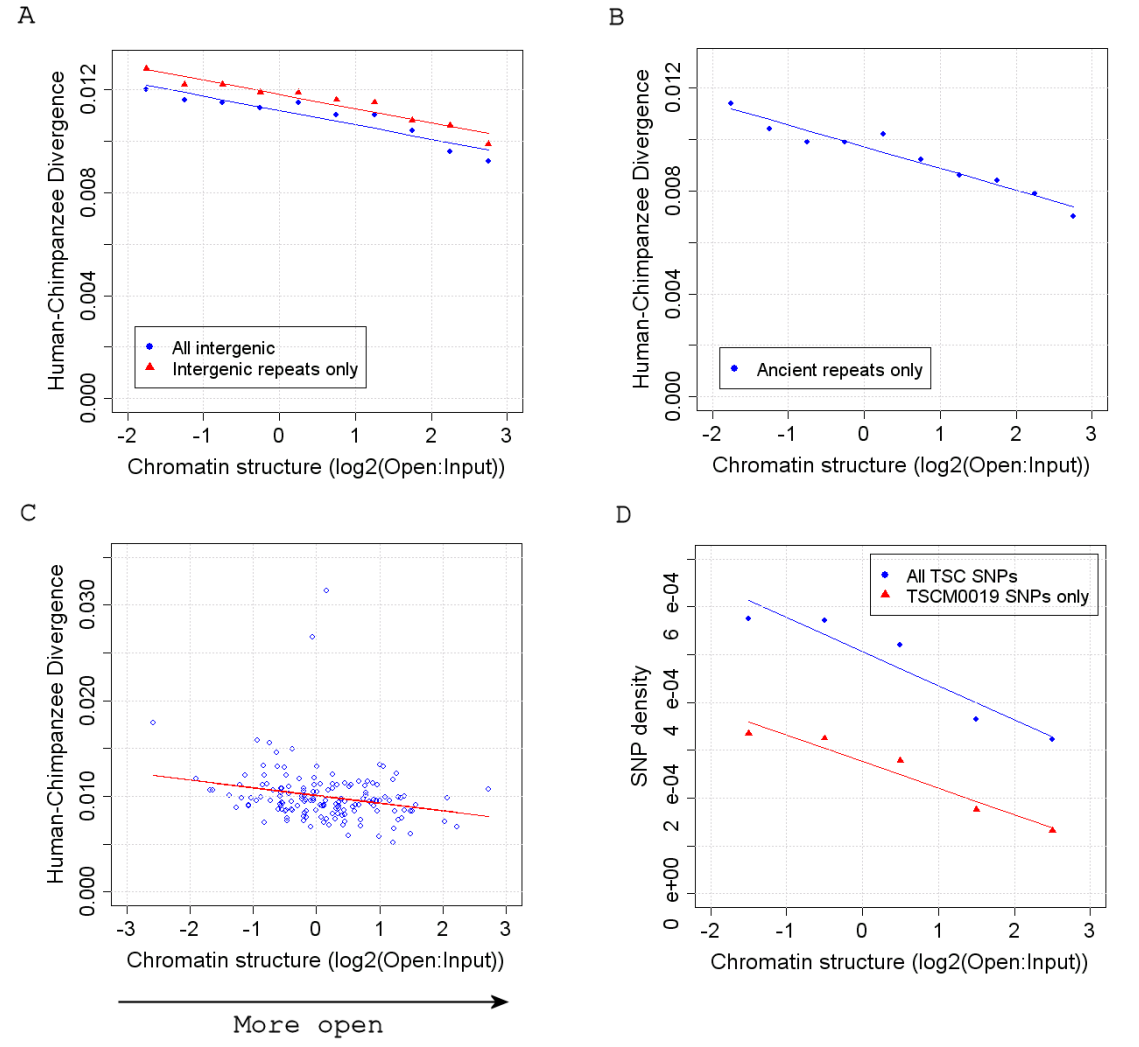
**Increased mutation rates in closed chromatin at non-CpG sites**. (A+B) Mean intergenic and ancient repeat divergence observed across chromatin categories (Intergenic r^2^: = 0.87, p = 9.1e-05; Intergenic repeats only r^2^: = 0.93, p = 7.3e-06; Ancient repeats only r^2^: = 0.93, p = 6.1e-06). (C) Intergenic divergence of each 1 Mb clone from chromosome 1 against their corresponding chromatin score (10 clones containing less than 10,000 intergenic bases were excluded). (D) Mean human SNP densities (SNPs/bp) observed across chromatin categories (All SNPs r^2^: = 0.89, p = 0.016; Single random detection protocol (TSCM0019) SNPs only r^2^: = 0.93, p = 0.008).

In order to determine whether background mutation rates are associated with chromatin structure we first determined intergenic divergence rates, using human versus chimpanzee whole genome alignments, in regions whose chromatin environment in human lymphoblastoid cells had been determined. The majority of intergenic bases should be under little or no selection and therefore intergenic divergence should be approximately analogous to background mutation rates. As shown in Figure 1A, we found a negative correlation between intergenic divergence and chromatin structure at non-CpG sites. As open chromatin is generally more gene rich than closed (and may therefore contain more regulatory elements than intergenic regions) we also examined divergence rates in ancient repeats only. However, these also displayed the lowest divergence rates when in open chromatin (Figure 1B).

It has previously been proposed that DNA sequences nearer the centre of the nucleus may be protected from DNA damage by those on the periphery (the "bodyguard hypothesis"). Likewise, the chromosomes most enriched with open chromatin are generally situated towards the centre of a nucleus [2]. The correlation observed between divergence rates and chromatin structure may therefore be an indirect result of these phenomena. We therefore investigated whether a correlation between intergenic divergence and chromatin structure could be observed within chromosomes. Although chromosomes themselves have been shown to display some level of polar organization (such that their most gene-poor regions are those closest to the nuclear periphery [7]) adjacent intergenic regions within chromosomes often have very different chromatin structures despite displaying approximately the same nuclear localisation. If the observed correlation between intergenic divergence and chromatin structure reflects the predictions of the bodyguard hypothesis we would expect to see no such correlation within chromosomes. This, however, is not the case. For example, as shown in Figure 1C, there is a significant negative correlation between intergenic divergence and chromatin structure within chromosome 1 (r^2 ^= 0.053; p = 0.0043). The two outlier clones observed in this figure, with a divergence greater than 0.025, could represent mutational hotspots in the genome. However, the degree of difference between the divergence observed in these clones compared with the rest of the chromosome suggests to us that the alignments in these regions are more likely be of poor quality. Removal of these clones increases the significance of the correlation observed between divergence and chromatin structure (r2 = 0.113; p = 2.5e-05). In total 7 out of 22 chromosomes display a significant negative correlation (p < 0.05) between clone intergenic divergence and chromatin structure (Chromosomes 1, 2, 5, 8, 12, 17 and 20). These data therefore argue against the bodyguard hypothesis being solely responsible for these observed correlations between chromatin structure and intergenic divergence rates.

Another measure often used to predict mutation rate is SNP density [8,9]. It is predicted that as a large proportion of intergenic sequence is non-functional and that there has been little time for selection to act on SNPs, their density along the genome should generally reflect underlying mutation rates. A further benefit of the use of SNPs in this way is that mutation rates can be predicted without relying on sequence comparisons with other species. We consequently determined the mean intergenic SNP densities observed across chromatin categories. As shown in Figure 1D the mean SNP density was also lowest in the most open regions of the genome.

**Figure 2:**
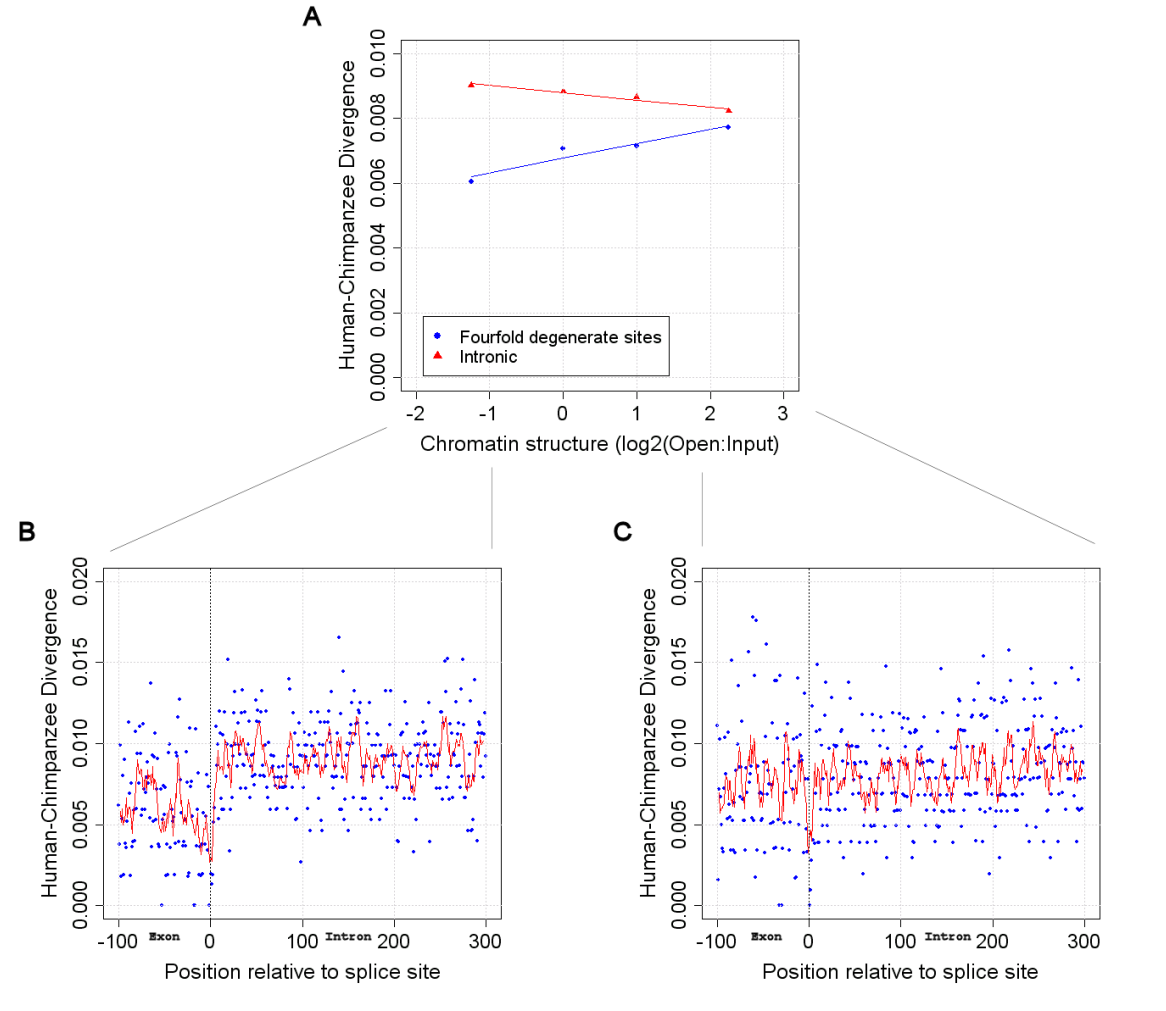
**Comparison of splice site divergence observed across chromatin categories**. (A) The divergence at non-CpG fourfold degenerate and intronic sites on autosomes only, with the divergence observed across the splice sites of the most closed (B) and open (C) genes shown below. (Closed exonic vs. closed intronic Mann-Whitney U test: p = 4.4e-16; open exonic vs. open intronic Mann-Whitney U-test: p = 0.053)

There is therefore strong evidence that mutation rates are associated with chromatin structure. Not only are intergenic, intronic (Figure 2A) and ancient repeat divergence rates highest in closed chromatin but the density of SNPs is also elevated in the most closed regions of the human genome. Thus we hypothesise that closed regions of the genome are simply less accessible to DNA repair mechanisms.

It should be noted however that chromatin structure is likely to be only one of several factors associated with neutral divergence rates in the human genome. This is most apparent on chromosome 19, and to a lesser extent chromosome 8, which show substantially higher mean intergenic divergence rates in our analysis than the other autosomes. Whereas chromosome 19 and chromosome 8 display mean intergenic divergences of 1.5% and 1.3% respectively, the divergence rates of all other autosomes fall between 1 and 1.2%. As chromosome 19 is particularly enriched with open chromatin [1], its high divergence levels are contrary to what is generally observed across the autosomes. The high levels of divergence observed along chromosome 19 are consequently likely to be a result of factors other than chromatin structure.

### Gene distribution and chromatin structure

**Figure 3:**
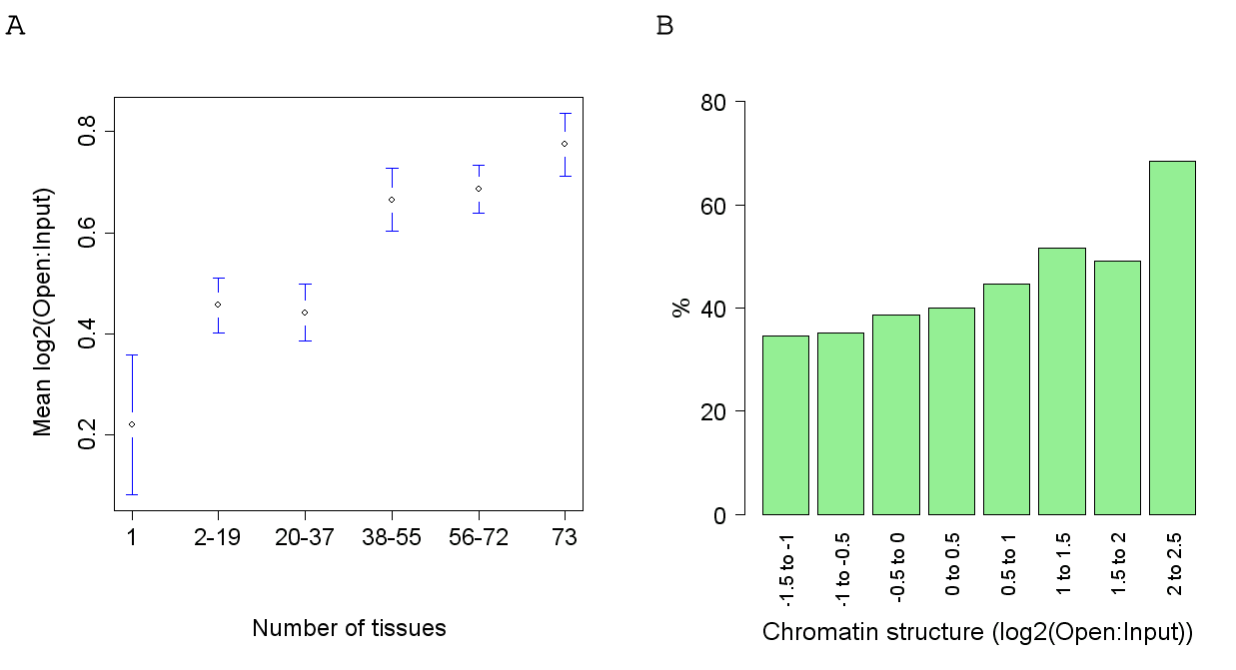
**The distribution of gene expression profiles and CpG island genes across chromatin categories**. (A) The mean chromatin structure (log2(Open:Input)) of genes of differing expression breadth across normal tissues (Kruskal-Wallis p = 7.5e-6) (B) The percentage of genes across chromatin categories that are associated with a CpG island.

As shown in figure 3A, housekeeping genes are generally located in the more open regions of the genome and tissue-specific genes in the most closed regions. This is in agreement with a previous analysis that illustrated that nucleosome formation potential is negatively correlated with expression breadth [10]. Consequently a recent study by Gazave et al. [11], that showed that the levels of human-chimpanzee divergence observed in the introns of housekeeping genes is significantly lower than in other genes, is in broad agreement with the analysis presented here. Although CpG dinucleotides were not excluded in Gazave et al's analysis this is only likely to have led to an increase in the estimation of divergence in housekeeping genes due to the enrichment of CpG dinucleotides in open chromatin. However, intriguingly, when human-mouse alignments are examined, the introns of tissue-specific genes have been shown to contain a greater proportion of conserved sequence than those of housekeeping genes [12] (in contradiction to what is observed in human-chimpanzee alignments). We believe this apparent discrepancy is likely to be the result of the difference in evolutionary distance investigated, with the examination of human-mouse alignments potentially leading to the identification of regions under (stabilising) selection. For example, we may expect that closed regions of the genome contain more DNA elements involved in regulating the surrounding chromatin structure whose conservation becomes apparent across larger evolutionary distances.

Through the use of the DAVID program [13] that determines those biological terms and annotations (for example GO terms) enriched among a set of genes, we identified further classes of genes most over-represented in closed chromatin, and therefore likely to be experiencing the highest mutation rates. Of the 148 genes in the most closed regions of the genome, 40 encode glycoproteins (p for enrichment: 0.000074) and 22 were associated with the G-protein coupled protein signaling pathway (p = 0.00011). Glycoproteins and G-protein coupled receptors are involved in immune response and cell signaling and it has previously been proposed that such genes are likely to evolve quickly in response to changing stimuli [4]. Being located in closed regions of the genome (where we have observed background mutation rates (intergenic divergence and SNP density) are particularly high) will allow this more rapid evolution. Housekeeping genes, on the other hand, that are enriched in open chromatin, have previously been shown to evolve relatively slowly [14]. The location of a gene in the genome and its subsequent local chromatin structure may therefore, at least partly, be governed by the suitability of the local mutation rate it confers.

### dS, unlike dN and dN/dS, is highest in regions of open chromatin

**Figure 4:**
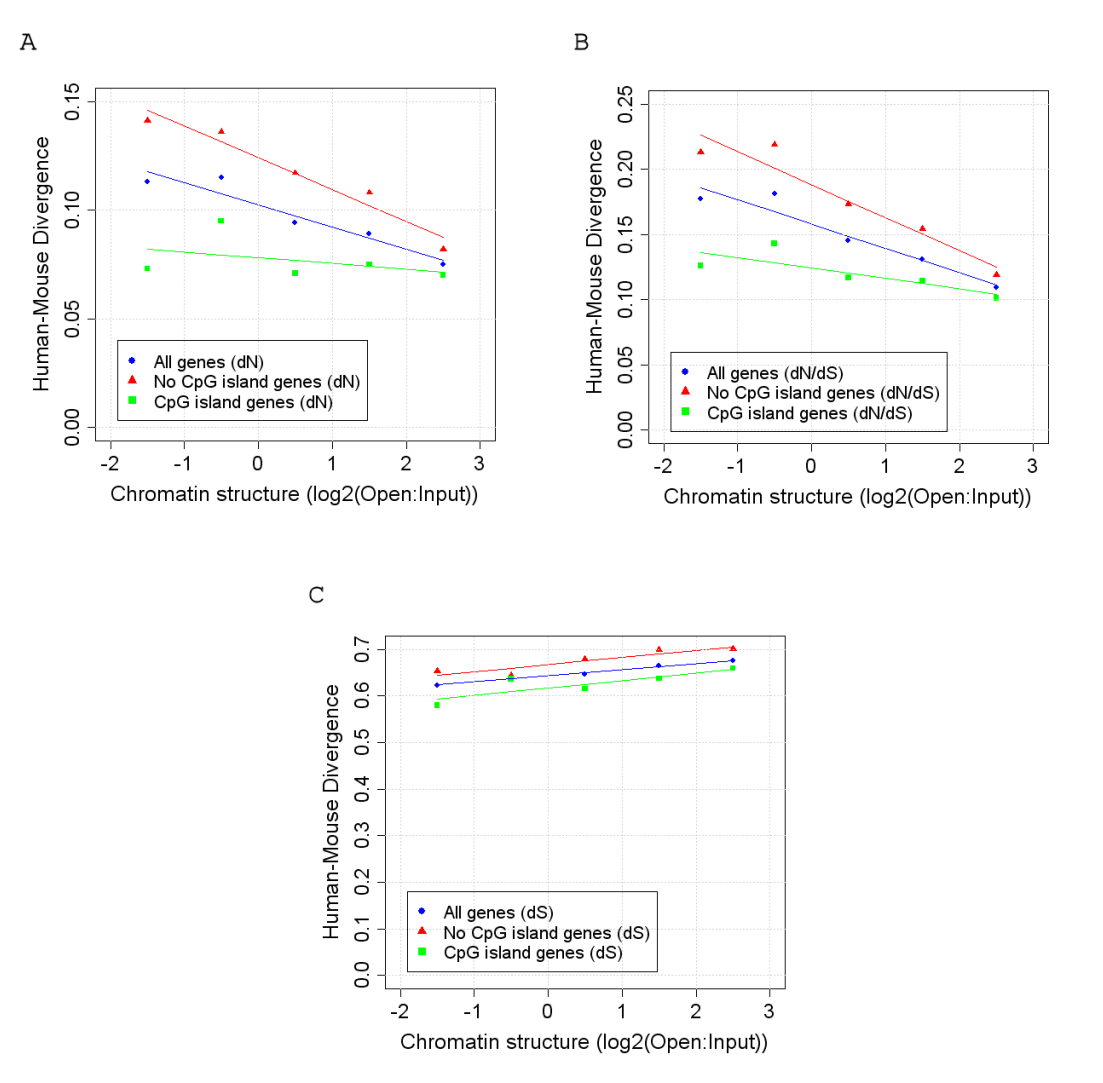
**Human-mouse divergence across chromatin categories**. Mean dN (A), dN/dS (B) and dS (C) in human/mouse coding sequence alignments. (All protein coding genes dS r^2^: = 0.99, p = 0.001; dN r^2^: = 0.92, p = 0.01; dN/dS r^2^: = 0.92, p = 0.009. Genes associated with a CpG island dS r^2^: = 0.72, p = 0.07; dN r^2^: = 0.17, p = 0.5; dN/dS r^2^: = 0.64, p = 0.1. Genes not associated with a CpG island only dS r^2^: = 0.84, p = 0.03; dN r^2^: = 0.95, p = 0.005; dN/dS r^2^: = 0.92, p = 0.01.).

dS has historically been used as a further surrogate measure of basal mutation rates, as synonymous sites were believed to be under little or no selection. Changes at synonymous sites, unlike at non-synonymous sites, do not affect the encoded amino acid. In addition, due to the relatively small effective population sizes of mammals, a synonymous site would have to experience relatively strong selection to evolve in a non-neutral manner [15]. As shown in Figure 4A, the average rate of non-synonymous changes (dN) observed in human mouse alignments is 51% higher in the most closed chromatin regions of the genome than in the most open regions. Similarly, the ratio of non-synonymous to synonymous substitution rates (dN/dS), which is frequently used as a measure of selection, is 61% higher (Figure 4B). However, the average synonymous rate (dS) for genes in relatively open chromatin is higher than that for genes in a more closed chromatin structure (Figure 4C). This is consistent with the reported high Ks for human chromosome 19, the human chromosome with one of the most open chromatin structures of all [16]. The observation by Hurst et al. of similar levels of human-mouse dS, dN and dN/dS in linked genes is likely therefore to be the result of linked genes being from similar chromatin environments. To ensure the converse is not true, and that the results observed in this study are not the result of linked genes, we randomly selected only one gene from each clone (so that all genes analysed were approximately 1 Mb apart and therefore unlinked). With this selection strategy we still observed similar correlations to those shown in Figure 3 (not shown).

Although we would expect the enrichment of housekeeping genes in relatively open regions of the genome as shown in Figure 3A (as open chromatin is likely to provide a more conducive environment for transcription), the lower average dN/dS observed in open chromatin may simply be a consequence of this higher number of housekeeping genes (which are known to evolve slowly) in these regions. The exclusion of housekeeping genes from the analysis, however, has little effect on the correlations in Figure 4 (not shown). Even the exclusion from the analysis of all genes whose 5' end is associated with a CpG island (which includes almost all housekeeping genes [17] and that are also enriched in open chromatin, Figure 3B) does not lead to the loss of the correlations between chromatin structure and dN, dS and dN/dS. In fact the rate of dN in CpG island genes, unlike that in genes not associated with a CpG island, is relatively constant across chromatin categories and does not show a significant correlation with chromatin. Consequently selection appears to maintain similar levels of dN in genes associated with a CpG island irrespective of their local chromatin structure.

To ensure these results were not confounded by CpG associated or sex chromosome specific factors (sex chromosomes have been shown to display abnormal rates of divergence when compared to the autosomes [18]), we calculated divergence rates at non-CpG, fourfold degenerate sites in genes on autosomes only. We also used human-chimp alignments instead of human-mouse alignments as the chromatin structure of the chimp genome should be more similar to that in humans (and consequently the species of origin for each change is less important). However, as shown in Figure 2A, the highest rates of divergence are still observed in genes from the most open regions of the genome.

### Genes in closed chromatin display the highest levels of selection at synonymous sites

Although historically the synonymous substitution rate (dS or Ks) has been used as a measure of the rate of mutation, there is increasing evidence that selection may be occurring at synonymous sites [15]. To investigate the potential role of any selection on synonymous sites in the disparity between dS and other measures of mutation, we analysed the rates of divergence observed across intron-exon boundaries [18]. As shown in Figure 2, the rates of intronic divergence in open regions of the genome are comparable to those observed at corresponding exonic, fourfold degenerate sites. This would suggest that genes in open chromatin display little if any evidence for selection at their synonymous bases. However, genes in closed chromatin display markedly higher rates of divergence at their intronic sites than at corresponding fourfold degenerate sites. Genes in closed chromatin therefore, unlike those in open, display strong evidence for synonymous site selection.

**Figure 5:**
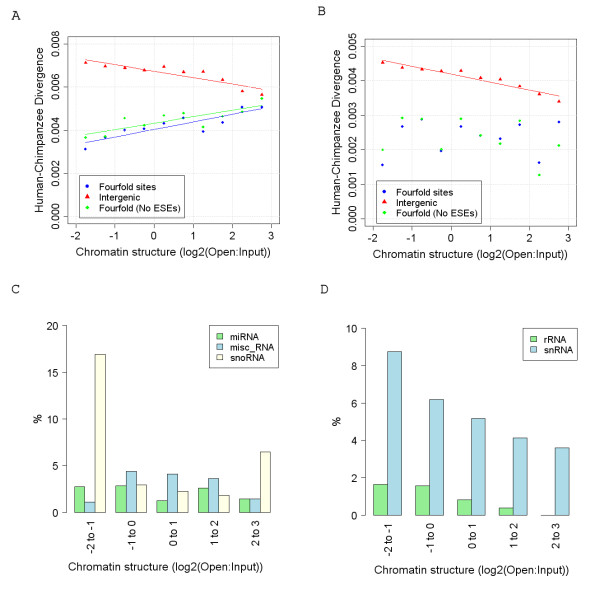
**The effect of ESEs on fourfold degenerate site divergence and the ncRNA gene distributions observed across chromatin categories**. (A+B) The observed rate of transitions and transversions respectively, at fourfold degenerate sites with and without ESE sites excluded (Fourfold degenerate site transversions r^2^: = 0.02, p = 0.69; fourfold degenerate site transversions at non-ESE sites r^2^: = 0.13, p = 0.30; intergenic transversions r^2^: = 0.92, p = 1.2e-05. Fourfold degenerate site transitions r^2^: = 0.78, p = 0.001; fourfold degenerate site transitions at non-ESE sites r^2^: = 0.67, p = 0.004; intergenic transitions r^2^: = 0.81, p = 4.0e-04). (C+D) The percentage of genes in each chromatin category that are of each Ensembl ncRNA class. Only the distributions of rRNAs and snRNAs show a significant negative correlation with chromatin structure (rRNA r^2^: = 0.96, p = 0.004; snRNA r^2^: = 0.92, p = 0.01)

Although the rate of selection against both synonymous transitions and transversions is highest in closed chromatin, only the rate of synonymous transitions is strongly positively correlated with chromatin structure (Figure 5A). The rate of transversions at fourfold degenerate sites shows no obvious trend across chromatin categories (Figure 5B) and consequently selection against transversions, unlike transitions, appears to be independent of any factors associated with chromatin structure. We are not aware of any reason for the observed association between rates of transitions at non-CpG fourfold degenerate sites and chromatin structure, but it could reflect constraint in motifs whose distribution are not uniform across the genome.

As previously shown, open regions of the genome are particularly gene dense whereas closed regions are relatively gene poor [1]. Consequently, the use of dS as a measure of mutation rate may be appropriate for a large proportion of genes. However, the use of dS as a surrogate measure of mutation rate for genes in closed chromatin will lead to the under-estimation of the true mutation rate in these regions and also the miscalculation of the levels of selection when used to measure dN/dS.

### Exonic Splice Enhancers and RNA secondary structure

It has been proposed that synonymous sites may experience constraint because they play a role in controlling splicing or RNA stability [15]. For example, synonymous sites may be part of an exonic splice enhancer (ESE) motif or lead to a more stable base-paired RNA that is less susceptible to degradation. Although codon usage bias (resulting from unequal abundances of tRNAs and subsequent selection at synonymous sites in favour of codons corresponding to the most abundant tRNAs) has also been proposed as an explanation of synonymous site selection, the evidence for this in mammals is weak [19]. We therefore looked at the distribution of each predicted ESE motif across chromatin categories to see if their relative densities could explain the high levels of synonymous selection in closed chromatin. The density of a large proportion of ESE hexamers (44%) displayed a significant negative correlation with chromatin structure. However, given the base composition of ESE hexamers and coding regions across chromatin categories, we actually observed far fewer hexamers displaying a negative correlation than we would expect by chance (66%). This is because coding sequence base composition is itself correlated with chromatin structure and ESEs also show biases in their base composition. As shown in Figure 5A, excluding all sites from coding regions that overlap a predicted ESE hexamer leads to only a small increase in the rate of transitions observed at fourfold degenerate sites. Consequently, either there are many ESE motifs that are yet to be determined, or selection at synonymous sites is at most only partly the result of exonic splice enhancers.

We also compared the distribution of gene types across chromatin categories. If genes whose RNA structure is important were preferentially located in closed chromatin we may expect an over-representation of non-protein coding genes in closed regions. As shown in figures 5C+D, certain classes of non-protein coding genes are indeed over-represented in closed chromatin (rRNAs and snRNAs), while the distribution of other types of genes such as miRNAs and snoRNAs show no relationship with chromatin structure.

Further analysis is therefore required to determine why protein coding genes in closed regions of the genome display such comparatively high levels of selection at their synonymous sites. If it is indeed because of a requirement for a more stable secondary structure, then we may expect that the predicted stability of mRNAs from closed regions would be greater than those in open [20]. Future tests of this kind may help determine the reasons behind the enrichment of selection at synonymous sites in closed chromatin observed in this study.

## Conclusion

We have shown that rates of mutation (intergenic, intronic and ancient repeat divergence as well as SNP density) and synonymous selection are correlated with chromatin structure. Regions of open chromatin display the lowest mutation rates and the least constraint at the synonymous sites of genes. Consequently previous observations of mutational hotspots in the human genome, high mutation rates around classes of genes involved in extracellular communication, the low dN/dS observed in housekeeping genes and the clustering of genes with similar divergence levels can all also be associated with chromatin structure. These correlations are observed despite the relatively low resolution of the chromatin dataset. The average length of the clones used in this analysis was 146 kb but the average human exon is approximately a thousand times smaller than this. There is consequently a disparity between the DNA regions whose rate of change we are measuring and the regions whose chromatin structure is known. The ability to measure chromatin structure at a higher resolution in the future may help increase the strength of these observed correlations.

We believe the lower background mutation rate observed in open regions of the genome in this study is likely to be a result of these regions being more accessible to repair mechanisms. Indeed it is known that sites of transcription-coupled repair are clustered in the gene dense (and therefore) open chromatin regions of the genome [21], that chromatin remodelling is a precursor to DNA repair, and that efficient DNA lesion detection is associated with relaxed chromatin structures [22-24]. However, contrary to mutation rate, we believe it unlikely that chromatin structure mediates selection on synonymous sites directly. Rather, it is more likely that genes that display a high level of selection at their synonymous sites are preferentially located in closed regions of the genome. It may be that these genes in general require especially tight transcriptional regulation, with a consequence being they are less accessible for DNA repair.

Chromatin structure is likely, however, to be only one of a number of factors that are associated with the variance in divergence rates observed across the human genome. This is supported by the fact that the levels of intergenic divergence of chromosome 19 are substantially higher than other autosomes, despite being gene dense and relatively open in structure. Most notably, both the chromatin dataset used in this analysis, as well as nucleosome formation potential [10], have previously been associated with GC content. Although this agreement between the lymphoblastoid chromatin dataset used in this analysis and other more general datasets is reassuring, GC content has previously been associated with rates of mutation and selection. However, although the mechanisms underlying the appearance of GC variability and isochores along the human genome remain controversial, it has been proposed that they may be a result of selection for the structural requirements of DNA. For example, an increase in GC content has been associated with an increase in bendability of DNA and a decrease in curvature, properties associated with more open chromatin [25]. Further analysis is consequently required to determine the complex interplay between the various factors involved in rates of mutation and selection across the human genome.

## Methods

The abundance of open chromatin fibre structure in lymphoblastoid cells, at clones spaced approximately 1 Mb apart along the human genome, was determined as previously described [1]. Relative chromatin structure was represented in this analysis by log_2_(open chromatin:input chromatin) values (determined by cohybridising differentially labelled "open" and input chromatin fragments to a human genomic DNA microarray). A large log_2_(open:input) value in this analysis indicates a region enriched with open chromatin (see Gilbert et al. for further details). Clones with similar log_2_(open:input) values were binned for analysis (with bin sizes adapted to the amount of data available). The 2,787 human protein coding genes that mapped to each of these clones and their corresponding mouse orthologues were obtained from Ensembl (unique best reciprocal hits were taken where possible then reciprocal hits based on synteny). Coding sequence alignments of each of these orthologous pairs were derived via protein alignments (using the MUSCLE [26] and tranalign [27] programs). The codeml program of the PAML package [28] was used to calculate dN, dS and dN/dS using the F3 × 4 codon evolution model. Gene pairs with anomalously high dS values (> 1.270 i.e. twice the median dS of all human vs. mouse pairs) were excluded [29].

Gene expression breadth was determined through the analysis of the Gene Expression Atlas Affymetrix U133A dataset of Su et al. [30]. Intensity levels were averaged across arrays derived from the same tissue and all tumour derived arrays were excluded. A gene was defined as expressed if its mean signal level across all its corresponding probes exceeded that of the data set median [12]. To identify potential genes with CpG islands, the positions of predicted CpG clusters were obtained from the UCSC genome browser [31]. Of these islands, any that were less than 500 bp long, had a G+C content less than 55 or had a CpG to expected CpG ratio of less than 0.65 were excluded [32]. Those genes whose 5' end was within 2 kb of one of these islands were determined to be potential CpG island genes.

Human chimpanzee divergence was determined through the use of the chained and netted human-chimpanzee alignments available at the UCSC website (hg17-panTro1) [33]. Ensembl gene predictions were used to identify intronic, intergenic and protein coding regions. All exclusively intergenic and intronic regions found within clones were identified, and divergence measured in the corresponding sections of the human-chimpanzee alignment using PAML's baseml with the REV model [28]. Before calculating divergence all sequence from the same chromatin category was concatenated, in order to minimise the problems inherent in accurately measuring low divergence levels in regions of finite length. All bases that overlapped a CG dinucleotide in either species were removed from the alignments to conservatively calculate non-CpG rates of divergence [18].

Intergenic repeats were identified through UCSC's RepeatMasker annotation. Ancient repeats were defined as in Gibbs et al [29] and Taylor et al. [34] as repeats from the same RepeatMasker subfamily conserved between mouse and human in the same orientation. Simple repeats and regions of low complexity were excluded.

The SNP Consortium data were used to calculate SNP density across chromatin categories [35]. To ensure these densities were not biased as a result of the variety of protocols used to detect SNPs (some of which were chromosome specific), SNP densities across chromatin categories were also calculated using only SNPs randomly identified via the TSCM0019 protocol (a panel of 24 DNAs sequenced by the Sanger Centre, for more details see: [36]). The location of TSC SNPs was determined by mapping their ssIds to current rsIds via data available at dbSNP.

Predicted Exonic Splice Enhancer (ESE) hexamers were obtained from Fairbrother et al. [37]. The occurrence of each of these hexamers in the coding regions of each of the genes that mapped to a 1 Mb clone was determined. In order to identify the number of hexamers we would expect to detect by chance given the base composition of the genes and hexamers, we randomly shuffled the bases in each of the coding regions 100 times and recalculated the occurrence of each of the hexamers. The distribution of non-protein coding genes across chromatin categories was determined through Ensembl annotations.

## Authors' contributions

JGDP undertook initial study design, software implementation, statistical analysis and interpretation, and drafted the initial manuscript. NG and WAB determined the chromatin structure of the 1 Mb cloneset, participated in the study design and contributed to the final manuscript. HC, MGD and CAMS participated in the final study design, coordinated the study and contributed to the final manuscript.
